# Supplementary material for: Autonomous Sensory Meridian Response self-reporters showed higher scores for cognitive reappraisal as an emotion regulation strategy
Source: PeerJ. 2021 May 26;9:e11474. doi: 10.7717/peerj.11474 (PMC8164417; doi:10.7717/peerj.11474)
Supplement: Supplemental Information 1 [file peerj-09-11474-s001.docx]

**Supplementary material**

**Interest in the term “ASMR” over time**

We evaluated the interest in the term “ASMR” over time in social media using Google Trends tool for Youtube searches and also the interest in the term “Autonomous Sensory Meridian Response” over time in scientific research, using the Pubmed search tool (We did not use the term “ASMR” because this same acronym is also used in Health Sciences for “age-standardized mortality rates”).





**Fig. S.1 Interest in the term ‘ASMR’ over time, using Google Trends for Youtube searches (which analyses the amount of Youtube searches over time, in all the world). The values reflect annual mean search interest relative to the maximum value in a given region and period. A value of 100 indicates the maximum popularity of a term, while 50 indicates that a term is half as popular relative to the maximum value**. **The search was done in Feb 2021.**





**Fig. S.2 Amount of papers published per year using the term ‘Autonomous Sensory Meridian Response.’. The search was done in Feb 2021 using Pubmed search tool.**

**Habits of viewing ASMR videos**

Additionally to the emotion regulation questionnaire, we asked participants if they have seen ASMR videos. In the ASMR self-reporters group, 56% of people have seen ASMR videos, which contrasts with the 44% who have not. In the Non-ASMR self-report group, 38% have seen ASMR videos, and 62% have not (*Figure S.3*).


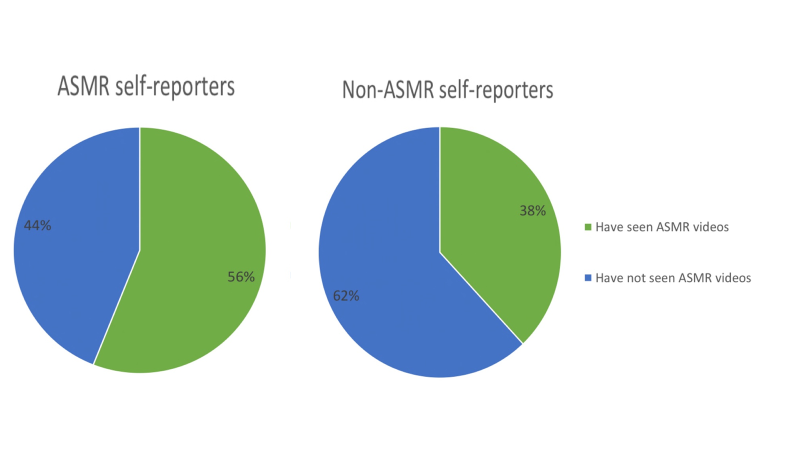


**Figure S.3 Percentage of participants in the ASMR and the non-ASMR group who have seen/listen to ASMR videos.**

Regarding the viewing/listening frequency of the ASMR videos, of the 39 participants in the ASMR group who have seen/listen to ASMR videos**,** 23% responded “every day”, 43% “at least once a week”, 31% “almost never” and only 3% responded almost “never” (*Figure S.4*).


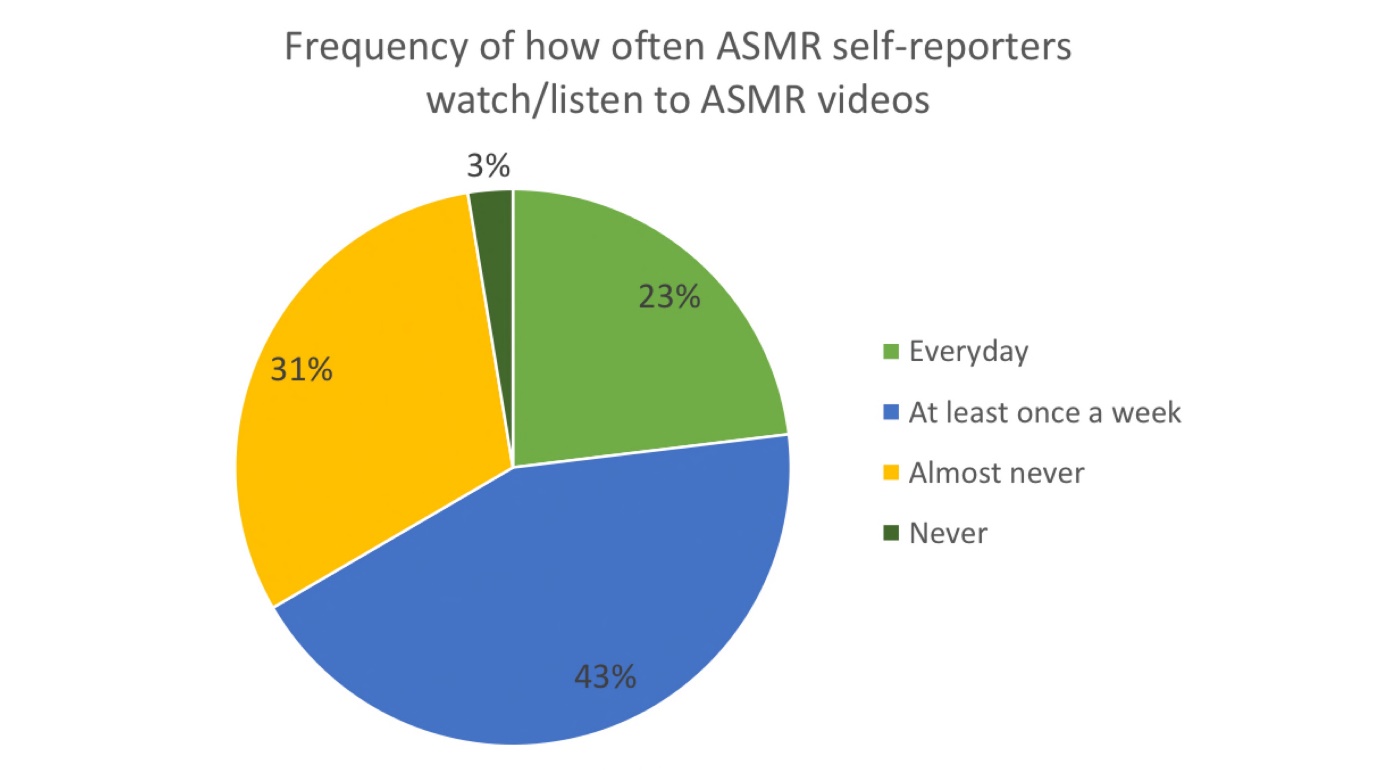


**Figure S.4 Frequency of how often ASMR self-reporters watch/listen to ASMR videos**

Finally, we assessed the main motivations of each group for watching ASMR videos. From 56% of ASMR self-reporters who watch ASMR videos (*Figure S.3*), the principal motivation for doing this is relaxation, followed by sleep induction and concentration while working/study. (*Figure S.5*). Interestingly, from 38% of the Non-ASMR group who watch ASMR videos, the principal reason is also relaxation, followed by curiosity (*Figure S.5*). As we could expect, the tingle induction is a motivation only present in the ASMR self-reporters group. However, it is not one of the main reasons for watching ASMR content.

**Figure S.5 Percentage of participants who watch ASMR videos based on their main motivations; relaxation, sleep induction, concentration, curiosity, tingle induction, or other reasons.**

**ASMR-15 Scale descriptive values**

**Table S.1 Descriptive comparison between the ASMR and non-ASMR group in the ASMR-15 scale**

|  | Group | | | | | |
| --- | --- | --- | --- | --- | --- | --- |
| Scale | ASMR | | | Non-ASMR | | |
|  | Mean | Median | SD | Mean | Median | SD |
| Sensation | 3.74 | 3.8 | 0.95 | 2.69 | 2.8 | 1.07 |
| Relaxation | 3.17 | 3.17 | 1.40 | 2.03 | 1.67 | 1.13 |
| Affect | 2.74 | 3.00 | 1.27 | 1.79 | 1.67 | 0.86 |
| Altered Conciousness | 2.51 | 2.50 | 1.14 | 1.86 | 1.50 | 0.96 |
| ASMR-15 total score | 3.10 | 3.07 | 0.96 | 2.16 | 2.23 | 0.7 |

**Multiple regression with gender as a predictor**

To evaluate if the gender of the participants had an effect on the association of ASMR and Cognitive reappraisal, we performed a multiple regression with the gender of participants as a predictor. For the analysis we included gender (Male = 1, Female = 0), self-reported ASMR (ASMR = 1, non-ASMR = 0) and subscales (Cognitive reappraisal = 1, Expressive suppression = 0) as predictors, with the scores of both subscales as the dependent variable. We also included the interaction between self-reported ASMR and scale, as it represents the main prediction of this article.

Two participants choose not to report their gender, so we exclude those subjects from the analysis. The sample included 103 female (51 in the ASMR and 52 in the non-ASMR groups) and 32 male (15 in the ASMR and 16 in the non-ASRM groups) participants. In neither group, participants with z scores larger than 3 or lesser than -3 were detected. Accordingly, we didn’t delete any case.

**Table S.2 Cognitive Reappraisal scores by gender**

|  | Group | | | |
| --- | --- | --- | --- | --- |
| Gender | ASMR | | Non-ASMR | |
|  | M | SD | M | SD |
| Male | 4.70 | 1.32 | 4.04 | 1.40 |
| Female | 4.69 | 1.13 | 4.24 | 1.08 |

**Table S.3 Expressive Suppression scores by gender**

|  | Group | | | |
| --- | --- | --- | --- | --- |
| Gender | ASMR | | Non-ASMR | |
|  | M | SD | M | SD |
| Male | 3.98 | 1.22 | 4.31 | 1.46 |
| Female | 3.40 | 1.30 | 3.55 | 1.50 |

*Table S.2* and *Table S.3* shows the scores by gender in the cognitive reappraisal and expressive suppression subscales. *Table S.4* shows the results of the multiple regression. After including gender (*B* = 0.29, *p* = .12) as a predictor the interaction between ASMR and cognitive reappraisal was significant (*B* = 0.73, *p* = .02), which showed significantly higher scores for the ASMR group in the cognitive reappraisal subscale (the main result of the article). Nevertheless, this result must be read with caution because the sample was mostly composed of female participants and, using all of the sample, gender was a significant predictor.

**Table S.4 Multiple regression of Emotion Regulation scores.**

| Variable | Coefficient | Standard Error | P |
| --- | --- | --- | --- |
| Constant | 3.67 | 0.16 | < .001** |
| Male | 0.29 | 0.19 | .12 |
| Cog. Reapp. | 0.46 | 0.22 | .04* |
| ASMR | -0.19 | 0.22 | .39 |
| ASMR X Cog. Reapp. | 0.69 | 0.32 | .03* |
| $R^{2}$ | .10 |  | < .001** |

Notes: N = 134, * *p* < .05, ** *p* < .001

**Analysis with all the sample**

To see if our results will be the same with all the samples, we performed the same analysis using all 176 subjects who responded to the survey (after excluding one subject who respond the same value in both emotion regulation subscales). The ASMR group was composed of 108 subjects (83 female, mean age =22.9, *SD* = 3.44), and the Non-ASMR group was composed of 68 subjects (52 female, mean age =23.81, SD = 5.51). Groups did not differ in age (*t*(174) = 1.35, *p* = .18) or in the proportion of male to female participants ($\chi^{2}$ (1) = 0.45, *p* = .83) in each group.

To test the assumptions of normality and homogeneity of variance, we use the Shapiro-Wilk and Levene tests, respectively. In the Expressive suppression subscale, data from the ASMR group showed a non-normal distribution (*W* =0.97, *p* = .03), and data from the non-ASMR group was normally distributed (W =0.97, p = .06). In the Cognitive reappraisal subscale, both the ASMR group (*W* =0.99, *p* = .25) and the non-ASMR group (*W* =0.98, *p* = .37) showed a normal distribution. The variance between the ASMR and non-ASMR groups were similar in both the Expressive Suppression subscale (*F*(1,174) = 1.56, *p* = .12) and in the Cognitive Reappraisal subscale (*F*(1,174) = 0.05, *p* = .82). As most of the data followed a normal distribution and had equal variances, we used an independent t-test to compare groups.

The ASMR group showed significantly higher scores in the cognitive reappraisal subscale than the non-ASMR group (*t*(174) = 2.34, *p* =.01, Cohen’s *d* = .36) (see *Table S.5*). There were no significant differences between ASMR and non-ASMR groups for the expressive suppression subscale (*t*(174) = -1.21, p = .22).

**Table S.5 ASMR and Non-ASMR scores in the Emotion Regulation Questionnaire**

|  | Group | | | | | |
| --- | --- | --- | --- | --- | --- | --- |
| Scale | ASMR | | | Non-ASMR | | |
|  | Mean | Median | SD | Mean | Median | SD |
| Cognitive Reappraisal | 4.59 | 4.67 | 1.08 | 4.19 | 4.17 | 1.16 |
| Expressive Supression | 3.46 | 3.75 | 1.34 | 3.73 | 3.5 | 1.51 |

To evaluate if the gender of the participants had an effect on the association of ASMR and Cognitive reappraisal, using all of the sample, we performed a multiple regression with the gender of participants as a predictor, with the same re-coding of the variables and interactions as the previous multiple regression.Table S.6 shows the same analysis presented in table S.3, but with all the samples (from a total of 176 subjects, three subjects who choose not to report their gender). Gender was a significant predictor (*B* = 0.35, *p* = .03), showing a tendency that males had a higher score in both subscales (see table S.7 and S.8). The interaction between ASMR and cognitive reappraisal was significant (*B* = 0.66, *p* = .02), which showed significantly higher scores for the ASMR group in the cognitive reappraisal subscale (the main result of the article), after including gender as a predictor. Nevertheless this results should be interpreted with caution, as the sample contained a much higher number of female participants.

**Table S.6 Multiple regression of Emotion Regulation scores, with the complete sample.**

| Variable | Coefficient | Standard Error | P |
| --- | --- | --- | --- |
| Constant | 3.65 | 0.16 | < .001** |
| Male | 0.35 | 0.17 | .03* |
| Cog. Reapp. | 0.46 | 0.22 | .03* |
| ASMR | -0.26 | 0.20 | .19 |
| ASMR X Cog. Reapp. | 0.66 | 0.28 | .02* |
| $R^{2}$ | .12 |  | < .001** |

Notes: N = 173, * *p* < .05, ** *p* < .001

**Table S.7 Cognitive reappraisal scores by gender, with the complete sample**

|  | Group | | | |
| --- | --- | --- | --- | --- |
| Gender | ASMR | | Non-ASMR | |
|  | M | SD | M | SD |
| Male | 4.69 | 1.28 | 4.04 | 1.40 |
| Female | 4.55 | 1.04 | 4.24 | 1.08 |

**Table S.8 Expressive Suppression scores by gender**

|  | Group | | | |
| --- | --- | --- | --- | --- |
| Gender | ASMR | | Non-ASMR | |
|  | M | SD | M | SD |
| Male | 3.99 | 1.02 | 4.31 | 1.46 |
| Female | 3.32 | 1.40 | 3.55 | 1.50 |
